# Development of a Standardized Semantic Feature-Based Reporting Proforma for Intraoperative Ultrasound Findings in Brain Tumors and Application in High-Grade Gliomas – A Preliminary Study

**DOI:** 10.1055/a-1637-9550

**Published:** 2021-11-17

**Authors:** Prakash Shetty, Vikas Kumar Singh, Amit Choudhari, Aliasgar V Moiyadi

**Affiliations:** 1 Tata Memorial Hospital, Neurosurgery, Mumbai, India; 2 Homi Bhabha National Institute, Health Sciences, Mumbai, India; 3 Tata Memorial Hospital, Radiodiagnosis, Mumbai, India

**Keywords:** intraoperative, brain, gliomas, semantic features, ultrasound

## Abstract

**Purpose** A semantic feature-based reporting proforma for intraoperative ultrasound findings in brain tumors was devised to standardize reporting. It was applied as a pilot study on a cohort of histologically confirmed high-grade supratentorial gliomas (Grade 3 and 4) for internal validation.

**Materials and Methods** This intraoperative semantic ultrasound proforma was used to evaluate 3D ultrasound volumes using Radiant DICOM software by 3 surgeons. The ultrasound semantic features were correlated with histological features like tumor grade, IDH status, and MIB index.

**Results** 68 patients were analyzed using the semantic proforma. Irregular crenated was the most common margin (63.2%) and lesions were heterogeneously hyperechoic (95.6%). Necrosis was commonly seen and noted as single (67.6%) or multiple (13.2%) in over 80% cases. A separate perilesional zone, which was predominantly hyperechoic in 41.8% and both hypo and hyperechoic in 12.7%, could be identified in 54.5% of cases. Grade 4 tumors were more likely to have an irregular crenated margin (71.2%) with a single large area of necrosis, while Grade 3 tumors were likely to have smooth (31.3%) or non-characterizable margins (31.2%) with no or multiple areas of necrosis. IDH-negative tumors were more likely to have a single large focus of necrosis. Among the GBMs (52 cases), MIB labelling index of>15% was associated with poorly delineated, uncharacterizable margins, when compared with MIB labelling index<15% (23.5 vs. 0%), (p=0.046).

**Conclusion** A detailed semantic proforma was developed for brain tumors and was internally validated. A few ultrasound sematic features were identified correlating with histological features in high-grade gliomas. It will require further external validation for refinement and acceptability.

## Introduction

Intraoperative ultrasound (iUS) has emerged as a robust and reliable tool for evaluating resection control in diffuse gliomas. The interpretation of tumor extent is the key variable influencing intraoperative decision making 1 2 3 . Image acquisition as well as image interpretation remains subjective. This subjectivity is one of the main hurdles in the widespread deployment of iUS. Practical training can minimize the problems with image acquisition 4 . Image interpretation too can be improved by various atlas-based and simulation techniques 5 . However, there is no uniformity in the descriptive terminology for intracranial ultrasound image reporting. In other areas (extra-cranial) ultrasound semantics have been streamlined to a large extent (breast – US-BI RADS; lung US semantics features, etc.). Unlike MRI, US image generation is based on echo properties of the tissues and hence MR-defined tumor semantic features may not be extrapolated accurately in this setting. A standardized semantic vocabulary for US is also desirable to ensure uniformity across reports and studies. Scattered studies in the literature describe sporadic semantic features with iUS for intracranial tumors. In this study, we reconcile these varied terminologies and propose a comprehensive semantic feature descriptor list for iUS examination of brain tumors, specifically gliomas. Furthermore, the semantic features were correlated with histological criteria to look for the predictive value of the semantic criteria in a cohort of high-grade gliomas. We present these findings for the first time.

## Materials and Methods

Development of the semantic feature-based proforma: The semantic ultrasound criteria for brain tumor insonation was designed to capture US data in a systematic way. The authors thoroughly reviewed the existing literature and identified multiple previous studies describing various ultrasound features in brain tumors 6 7 8 9 10 11 . The VASARI (Visually AcceSAble Rembrandt Images) criteria used for standardized reporting of brain tumors on MRI were referenced as a guide 12 . Using a combination of various previously described features along and new features extrapolated from the VASARI schema as well as from experience of the authors, a comprehensive semantic-based ultrasound feature list was developed. This form essentially captured the following information:

1. Ultrasound image quality
2. Normal anatomical structure identification
3. Brain tumor interface
4. Lesion delineation
5. Intralesional US features including echotexture, uniformity, presence of necrosis, cyst, calcification besides others.
6. Perilesional zone features - margin, extent, and echotexture.

The detailed proposed proforma is attached in **Appendix 1** ( **Supplementary Material** ). Multiple drafts of the proforma were discussed internally and a final version was approved for testing based on a consensus decision.

The proforma was applied as a pilot study to a cohort of histologically confirmed high-grade supratentorial gliomas (grade 3 and 4). The study was approved by the institutional ethics committee (IEC-ACTREC 209). 3D ultrasound data was captured prospectively in patients undergoing navigated ultrasound-guided resections using Sonowand (SONOWAND AS, Trondheim, Norway). Informed consent was obtained from all patients. These images were then analyzed for this study. At the time of acquisition, surgeons attempted to obtain the best quality and as large a field of view of the images as possible. One of the surgeons (AM) reviewed all of the 3D volumes for overall adequacy and these were then anonymized to minimize bias for further review. No clinical or radiological (MRI) data was provided during the reviewing process. The anonymized 3D ultrasound volume data was reviewed by three neurosurgeons (AM, PS, VS) sitting simultaneously. The images were reviewed using Radiant DICOM Viewer (company-Mexidant, Version 2020.2.3 ). The loaded 3D volume was reformatted in the axial, sagittal, and coronal planes using the multiplanar reconstruction function for the review process. The semantic criteria were discussed jointly and recorded as per the proforma. Any differences in opinion were resolved by consensus to account for interrater variability (due to the subjective nature of a few semantic criteria) which can be a confounding factor. The data obtained was correlated with existing histopathological variables like

1. Tumor grade: The tumors were classified according to WHO 2016 histological grading (grades 1–4). The tumors that were classified as impending GBM were classified as grade 4 tumors.
2. IDH status: As a routine, IHC is performed for IDH status and sequencing is used to confirm only negative or equivocal cases. For the analysis, IDH status was dichotomized as positive (grade 3 and secondary GBMs) and negative (primary GBMs).
3. MIB labelling index: The MIB labelling index was reported as a range in many cases and for the analysis the mean value was taken for an individual patient. An arbitrary cut-off value such as 15% was used as described in a previous study for analysis 11 .

Study statistics: The study database was maintained on SPSS (version 20.0.0). Frequency analysis was used to check the distribution of various parameters. The correlation between various categorical data was done using the Fischer’s exact test (p<0.05 was considered significant).

## Results

100 prospectively collected 3D US volume sets were available. After excluding low-grade gliomas (n=2) and non-glial tumors (21 cases), a total of 77 high-grade glioma cases were analyzed using the devised semantic form/classification. 9 (11.6%) image sets were discarded due to poor image quality. 68 patient data sets were available with complete radiological and primary histopathological data. IDH status and MIB labelling data were available for 57 and 66 patients, respectively.

### Semantic features in high-grade gliomas (n=68)

88.2% patients had a good quality scan, while 11.8% had a moderate quality scan. The lesion was easily discernable in 97.1% of patients. The entire lesion was visible in 97.1% of patients.

The margin delineation (brain-tumor) was good in 20.6%, moderate in 60.3%, and poor in 19.1%. The margin was further characterized in those cases where it was either good or moderately defined (55 cases). This margin was of two types: between lesion and brain interface (25 cases - when perilesional zone was absent) and between lesion and perilesional zone (30 cases - when it was present). The most common margin type was irregular crenated finger-like processes (63.2%), followed by regular lobulated (14.7%), whereas a regular smooth margin was infrequent (2.9%) in high-grade gliomas. In 13 cases with poor margin visualization and non-characterizable margins, 2 cases had a perilesional zone.

The lesion was hyperechoic in all cases, most being heterogeneously hyperechoic (95.6%) with varying internal echotexture areas. Cysts were identified in 23.5% of cases, with 7.4% having a single cyst and 16.1% having multiple cysts. All of the cysts were intralesional. Necrosis was commonly seen and noted as single (67.6%) or multiple (13.2%) in over 80% of cases, whereas it was absent in19.1%. When necrosis was reported,>50% area of necrosis was the most common finding in 58.8% of cases. Only 1 case of calcification was identified by US.

**Fig. 1:**
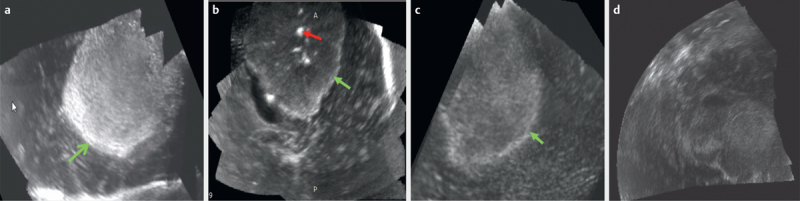
B-mode ultrasound image of a high-grade glioma showing **a** Regular smooth margin (green arrow) **b** Regular lobulated margin (green arrow) with calcification (red arrow) **c** Irregular crenated margins (green arrow) **d** Uncharacterized margin.

Perilesional zone: A separate perilesional zone could be identified in 54.5% of cases and was absent in 45.5%. It was predominantly hyperechoic in 41.8% and both hypo- and hyperechoic in 12.7% cases. The extent of the perilesional zone was less than the size of the tumor in 23.6%, equal to the size of the tumor in 12.7% and greater than the size of the tumor in 18.2%. Figures 1 2 3 4 5 depict the various semantic features described in this schema.

**Fig. 2:**
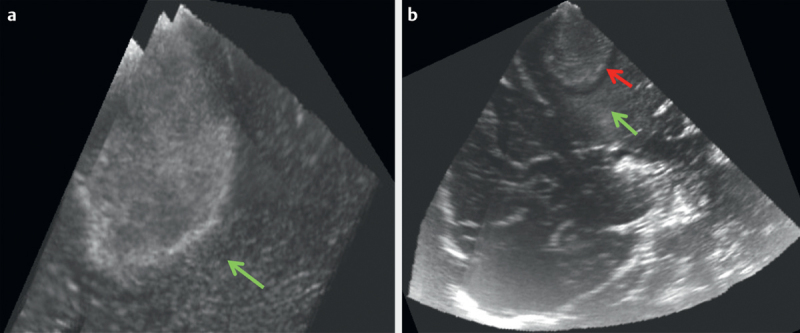
B-mode ultrasound image of a high-grade glioma showing **a** Hyperechoic perilesional zone (green arrow) **b** Hyperechoic (green arrow)+hypoechoic perilesional zone (red arrow).

**Fig. 3:**
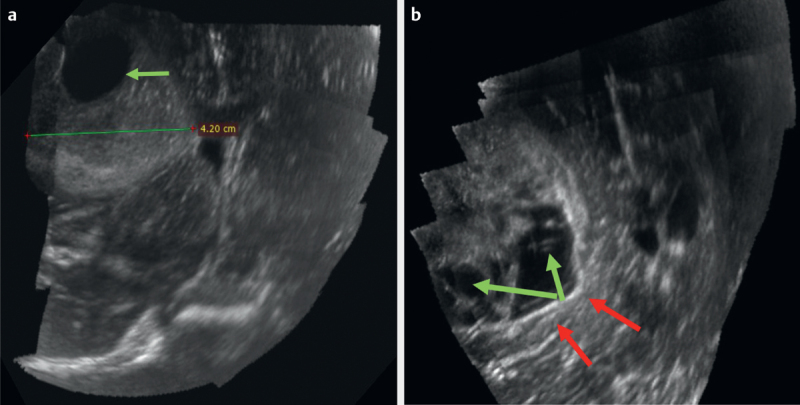
B-mode ultrasound image of a high-grade glioma showing **a** Single large intralesional cyst (green arrow) **b** Multiple intralesional cysts (green arrows) and posterior acoustic enhancement artifact (red arrows).

**Fig. 4:**
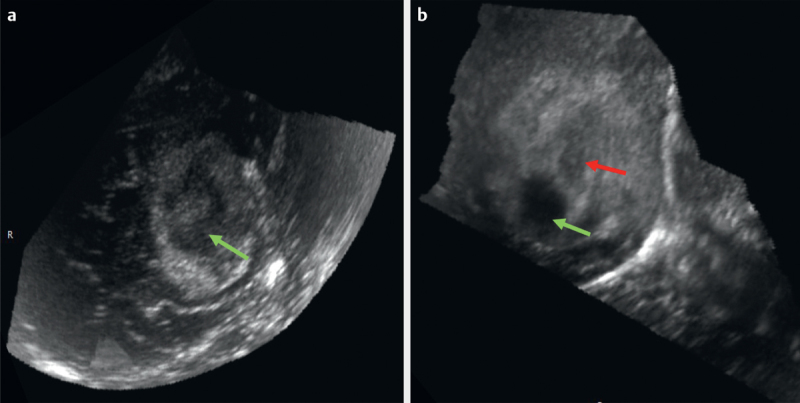
B-mode ultrasound image of a high-grade glioma showing **a** Necrosis with double density **b** Cyst (green arrow) with necrosis (red arrow).

**Fig. 5:**
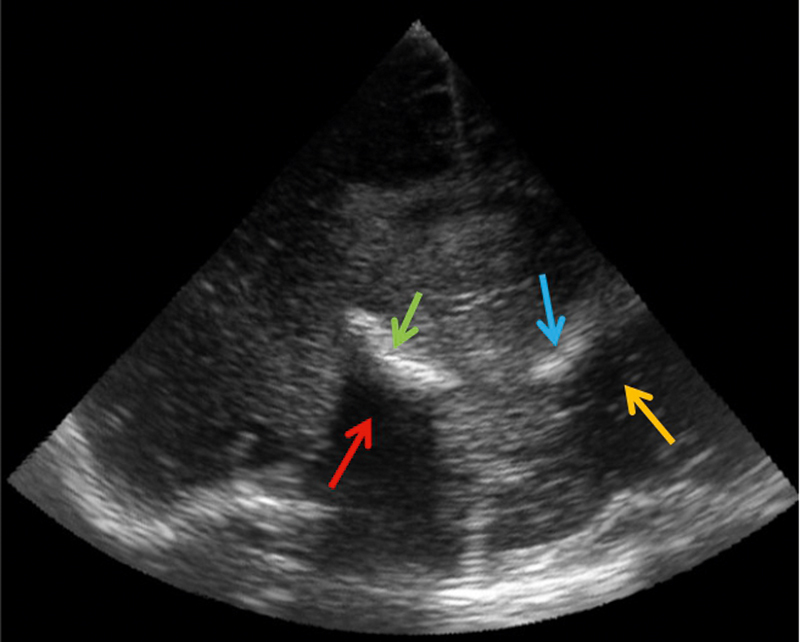
B-mode ultrasound image of a high-grade glioma showing acoustic shadow (red arrow) due to calcification (green arrow) and mirror artifact of the calcification (blue arrow) and shadow (yellow arrow).

### Correlation of semantic and histological features

Table 1 shows the distribution of histological types, grade, and IDH status of the analyzed tumors.

**Table:** **Table 1** Case distribution in various histological categories.

	Grade 3		Grade 4 (Glioblastoma – GBM)	
**Grade (68 cases)**	16 (23.5%)		52 (76.5%)	
**Histology (68 cases)**	Astrocytoma – 10 (14.1%)	Oligodendroglioma – 6 (8.8%)	Secondary GBM – 7 (10.3%)	Primary GBM – 45 (66.2%)
**IDH status (57 cases)**	IDH-positive – 7	IDH-positive – 6	IDH positive – 7	IDH-negative – 37

All of the semantic features were individually correlated with grade (3 or 4), IDH status (positive or negative), and MIB labelling index (<15% and>/=15%). The significant co-relations are mentioned in Table 2 and described below.

**Table:** **Table 2** Statistically significant correlation of ultrasound semantic features with histological features.

S. no.	US feature	Grade of tumor (68 cases)	IDH status (57 cases)	MIB labelling (66 cases)
1	Lesion visualization	P=0.05	X	X
2	US margin delineation	X	X	X
3	US margin type	P=0.014	X	X
4	Echotexture	X	X	X
5	Internal heterogeneity	X	X	X
6	Presence of cyst	X	X	X
7	Necrosis	P=0.001	P=0.025	X
8	Necrosis quantification	P=0.003	P=0.012	P=0.018
9	Calcification	X	X	X
10	Separate perilesional zone seen	X	X	X
11	Perilesional zone characteristic	X	X	X
12	Perilesional zone margin	X	X	X
13	Extent of perilesional zone	X	X	X

- **Lesion visualization** : All grade 4 tumors (100%) were easily discernable. 2/16 (12.5%) of grade 3 tumors were discernable with difficulty (p=0.05) ( Table 3 ).
- **Lesion margin** (brain-tumor interface) type: Grade 3 tumors were more likely to have regular margins (31.3 vs. 13.5% in grade 4 tumors), whereas grade 4 tumors are more likely to have irregular crenated finger-like margins (71.2 vs. 37.5% for grade 3 tumors) and this difference was statistically significant (p=0.014). However, it is to be noted that 31.2% of grade 3 tumors and 15.4% of grade 4 tumors had an uncharacterizable margin due to poor margin delineation ( Table 4 ). However, the perilesional zone by itself did not show statistical association with histological factors but did when associated with the margin type. Whenever the perilesional zone was absent, grade 3 tumors were associated with non-characterizable margins (5/10), while grade 4 tumors were associated with irregular crenated margins (17/24), p=0.019 ( Table 5 ). Non-necrotic tumors were predominant in grade 3 (4/5) while necrotic tumors were common in grade 4 (7/8), in patients with poor margin delineation (p=0.007).
- **Necrosis:** The presence of necrosis was statistically associated with tumor grade (p=0.001) and IDH status (p=0.025). Grade 4 tumors were more likely to show necrosis (89%) vs. grade 3 tumors (56.2%). When present, the necrosis in grade 3 was equally likely to be multiple small foci (25%) or a large confluent area (33%) as compared to grade 4 where it was overwhelmingly likely to be a single large confluent area (79%). A similar trend was seen for IDH-negative tumors (more necrosis (92%), and most of it being a single large confluent area – Table 6 ) as opposed to IDH-positive tumors which were less likely to have necrosis (70%, with both single (50%) and multiple areas (20%) seen).
- **Necrosis quantification:** The quantity of necrosis was associated with tumor grade (p=0.003), IDH status (p=0.012), and MIB labelling index (p=0.018). Grade 4 tumors are more likely to have necrosis that is more than 50% of the tumor volume as compared to grade 3 tumors (69.2 vs. 28.6%). Similarly, IDH-negative tumors are likely to have necrosis involving 50% of the tumor volume compared to IDH-positive tumors (73 vs. 36.8%). Similarly, tumors with an MIB labelling index of>15% are more likely to have a large necrotic area (>50% of tumor volume) compared to tumors with MIB≤15% (75 vs. 39.3%) **(** Table 7 ).

**Table:** **Table 3** Correlation of ultrasound visualization and grade of tumor.

	Grade 3	Grade 4
US visualization		
Easily discernible	14 (87.5%)	52 (100%)
Discernible with difficulty	2 (12.5%)	0 (0%)
	P=0.05	

**Table:** **Table 4** Correlation of ultrasound margins with grade of tumors.

	Grade 3	Grade 4
US margins		
Regular smooth	2 (12.5%)	0 (0%)
Regular lobulated	3 (18.8%)	7 (13.5%)
Irregular crenated	6 (37.5%)	37 (71.2%)
Cannot be characterized	5 (31.2%)	8 (15.4%)
	P=0.014	

**Table:** **Table 5** Correlation of US margin and perilesional zone with tumor grade (n=68).

	US margin (brain-tumor interface)				Total	
Perilesional zone		Regular smooth	Regular lobulated	Irregular crenated	Cannot be characterized		P-value
Yes	Grade 3	1	1	3	0	5	0.244
	Grade 4	0	5	20	2	27	
**No**	**Grade 3**	**1**	**2**	**3**	**5**	**11**	**0.056**
	**Grade 4**	**0**	**2**	**17**	**6**	**25**	

**Table:** **Table 6** Correlation of ultrasound-detected necrosis and grade of the tumor.

	Grade 3	Grade 4	IDH-positive	IDH-negative
Necrosis				
Absent	7 (43.8%)	6 (11.5%)	6 (30%)	3 (8.1%)
Single	5 (31.2%)	41 (78.8%)	10 (50%)	31 (83.8%)
Multiple	4 (25%)	5 (9.6%)	4 (20%)	3 (8.1%)
	P=0.001		P=0.025	

**Table:** **Table 7** Correlation of ultra-sonographic necrosis quantification with grade, IDH status, and MIB labelling index of the tumor.

	Grade 3	Grade 4	IDH-positive	IDH-negative	MIB (0–15%)	MIB>15%
Necrosis						
Absent	7 (43.8%)	6 (11.5%)	6 (30%)	3 (8.1%)	9 (31%)	4 (10.8%)
<10%	0 (0%)	1 (1.9%)	1 (5%)	0 (0%)	1 (3.4%)	0 (0%)
10–50%	5 (31.2%)	9 (17.3%)	6 (30%)	7 (18.9%)	8 (27.6%)	6 (16.2%)
>50%	4 (25%)	36 (69.2%)	7 (35%)	27 (73%)	11 (37.9%)	27 (73%)
	P=0.003		P=0.012		P=0.018	

Other features did not show statistically significant differences ( Table 2 ).

Among GBMs: (52 cases) – MIB labelling index of>15% was associated with poorly delineated, uncharacterizable margins, when compared with MIB labelling index<15% (23.5 vs. 0%), (p=0.046). ( Table 8 )

**Table:** **Table 8** GBM- correlation between US margin type and MIB labelling index (GBM cases 52, MIB data -50).

US margin	MIB 0–15%	MIB>15%
Regular lobulated	4 (25%)	3 (8.8%)
Irregular crenated	12 (75%)	23 (67.6%)
Cannot be characterized	0 (0%)	8 (23.5%)
	P=0.046	

## Discussion

The need to obtain information from imaging techniques has evolved over the last few decades. The development of the PACS (Picture Archiving and Communications system) helped with the storage of a large amount of radiological data in a filmless format. Since radiological data interpretation was subjective, attempts were made to provide meaningful and uniform reporting of data (semantics). Various standard reporting (semantics-based) format have evolved including VASARI (Visually AcceSable Rembrant Images) to describe MRI imaging findings in high-grade gliomas 12 and RadLex developed by the Radiological Society of North America (RSNA), which is a comprehensive radiology ontology tool, for uniform indexing of radiological information 13 . Large scale standardization and linkage of image data are also crucial and address the expanding need and scope of machine learning algorithms which are now an integral part of radiological image analysis and interpretation.

Ultrasound image analysis have relatively lagged behind as ultrasound is a real and image acquisition and interpretation are heavily operator-dependent. This leads to a lack of uniformity in reporting. Also, few centers archive real-time ultrasound, though most would store the relevant 2D images. Considering that most intraoperative cranial ultrasound examinations are performed by non-radiologists (neurosurgeons), there is a pressing need to standardize terminology and reporting formats while capturing the data. There have been attempts in the past by various groups to describe semantic features for reporting of intracranial ultrasound for brain lesions ( Table 9 **).** These authors have looked at various US features including visibility (discernibility), echogenicity, border visualization, and perilesional zone characteristic. The simple classification of tumors used in a study by Mair et al. may not capture the subtleties especially in the case of non-enhancing gliomas 6 . Specifically, the definition of the tumor border as binary “poor” and “good” may not be accurate to describe some gliomas with mixed US patterns. Studies by Wang et al. and Chen et al. also provided only descriptive observations and lacked more objective evaluation of the parameters 8 9 . However, there is no currently available structure that incorporates all of these ultrasound characteristics for comprehensive semantic reporting. Our attempt represents the first such comprehensive semantic classification system ( **Appendix 1** ).

**Table:** **Table 9** Studies describing the use of intraoperative ultrasonography in cranial lesions.

	Ultrasound features for classification of IC lesions	Conclusions
Mair et al. (2013)- 6	Classified based on visibility and demarcation 0 – Invisible on US 1 – Poor visibility and poor borders 2 – Good visibility and poor borders 3 – Good visibility and good borders	Glioblastomas, meningiomas, metastases, ependymoma, and hemangioblastomas were the best delineated group (median scores of 3). Interestingly, low-grade gliomas had a lower score of 1–2 compared to high-grade gliomas
Aeur et al. (1990)- 7	Used echogenicity, border delineation, & peri-tumoral edema. First to describe two types of perilesional zones (hypo- and hyper-echoic)	Provided one of the earliest descriptive overviews of various texture features
Wang et al. (2008)- 8	Described US features of 98 gliomas (grades 1–4) using echogenicity, border delineation, and surrounding edema as criteria	Reported low-grade gliomas to be well circumscribed with a regular shape and homogeneous echotexture without perifocal edema, as opposed to high-grade gliomas which were more heterogeneous with unclear margins. No objective quantification or correlation provided.
Chen et al. (2004)- 9	Described 37 intracranial masses (a heterogenous group with only 3 gliomas) primarily focusing on the echo characteristics and vascularity.	Reported low-grade astrocytomas to be isoechoic to hyperechoic, whereas high-grade gliomas were more heterogeneous with hypo-, iso-, and hyperechoic regions. No objective quantification or correlation provided.
Cengiz et al. (2005)- 10	Described US features of malignant tumors (a total of 40 gliomas and metastases).	Described internal necrotic cysts (with irregular shaggy margins and double-densities) in addition to irregular tumor contour and perilesional edema as hallmarks of malignant intra-axial tumors.
Baskan et al. (2015)- 11	Described a more objective US assessment of gliomas differentiating features of LGG from HGG (15 LGGs and 26 HGGs). They used echogenicity, margin, and peripheral edema as described by other authors. In addition, they defined contours as regular and irregular.	LGGs likely to be homogeneously hyperechoic with distinct margins and regular contours and no surrounding edema. They also dichotomized GBMs based on proliferative index (Ki-67 labelling of 15% as cutoff) and found that whereas the internal heterogeneity was present in both high (>15%) and low (<15%) proliferative groups, the low proliferative GBMs were more likely to have less hyperechoic solid areas, distinct margins, and regular contours.

Our results are similar to previous studies in which high-grade gliomas were likely to be more hyperechoic and heterogeneous compared to low-grade gliomas 8 9 11 . We did not apply this classification to low-grade gliomas and, therefore, cannot directly compare the features of low-grade gliomas with higher-grade ones. Objectively grading “ **echogenicity** ” is very difficult on ultrasound images especially if acquired on different machines with different gain settings.

A well-defined smooth margin usually denotes a low-grade lesion, and high-grade lesions that grow over a short period of time tend to have irregular crenated margins. Another type of margin is when the tumor grows slowly and merges with the surroundings very gradually (non-characterizable), sometimes also seen in low-grade tumors e. g., gliomatosis. In our study, the majority of grade 4 tumors had an irregular crenated margin, while a relatively large proportion of grade 3 tumors without a perilesional zone had diffuse non-characterizable margins. Previous reports suggest that high-grade gliomas were more likely to have ill-defined margins compared to low-grade tumors 8 11 . However, the study by Mair et al. showed low grade gliomas are more ill defined when comapred with capturing few low-grade tumors with diffuse boundaries 6 . We feel that there are two types of pattern of spread in gliomas: irregular crenated finger-like spread commonly seen in grade 4 gliomas and a diffuse non-characterizable growth pattern seen in grade 3 tumors, especially in diffuse tumors without a perilesional zone.

A perilesional zone was present in half the cases of high-grade gliomas in our series and were likely to be hyperechoic or mixed in the majority of cases. Auer et al. described the presence of a hyperechoic zone (considered edema) in 44 out of 73 gliomas studied by US, whereas in the remaining, no differentiation was possible 7 . Wang et al. also reported that the perilesional zone was more extensive and ill-defined in high-grade gliomas 8 . Chengiz et al. described the presence of an inner hypoechoic zone (ischemic and gliotic tissue) and an outer hyperechoic zone (due to malignant edema) in perilesional zones of malignant lesions 10 . We also had a few mixed cases as reported by them. Hence, we feel that it is more appropriate to call this a **peritumoral “zone”** and not “edema” acknowledging the fact that sometimes it may be difficult to differentiate edema from an infiltrating tumor. This hypoechoic zone is difficult to identify (and many times is truly absent) in some diffuse infiltrative tumors and hence is often not documented. Its exact significance is not clear. It could serve as a useful contrast (when present) between tumor and normal brain (the hypoechoic rim can accentuate the border zone making it more clearly visible). A second zone beyond this is described as being hyperechoic and could be a result of edema or tumor infiltration. Although edema is less hyperechoic than infiltrated brain, it may be difficult to clearly distinguish the two. If this zone is separated from tumor by a hypoechoic zone, then this is more likely to be edema.

We were able to show specific semantic features to differentiate grade 3 from grade 4 gliomas as well as IDH status. Also there was an overlap of features between grade 4 gliomas (which are predominantly IDH-negative) and IDH-negative lower-grade tumors and this may reflect our evolving understanding of the molecular similarity between these two groups. Grade 4 tumors appeared to have better discernibility and were more likely to have an irregular crenated margin with a single large area of necrosis as compared to grade 3 tumors. Similarly, IDH-negative lower-grade tumors were associated with a single large area of necrosis on ultrasound compared to IDH-positive tumors. IDH status has implications for overall survival (OS), with IDH-positive tumors doing better than negative. In lower-grade gliomas (grade 2–3), the impact of surgery on survival is greatest for IDH-positive tumors, especially astrocytoma. For IDH wild type lower-grade gliomas, the extent of surgery is not associated with OS 14 . However, we did not have any IDH-negative tumors in the grade 3 cohort.

For gliomas, an increased MIB index is associated with aggressive behavior and an increased risk of recurrence 15 . Tumors with an MIB labelling index of more than 15% were likely to have a single area of necrosis and necrosis>50% of area on ultrasound features when compared to a tumor with an MIB labelling index less than 15% in our study. Among GBMs, an MIB labelling index of>15% was associated with diffuse non-characterizable margins similar to the study by Baskan et al. 11 .

### Limitations and perspectives

We attempted to incorporate all possible features based on our experience and data from published literature. We also applied this preliminarily to a cohort of high-grade gliomas and found certain features correlating with the tumor grade and molecular type. The application of this classification system for low-grade gliomas needs to be ascertained in the future. Furthermore, in the present study, we used a consensus approach to record the findings. Considering the subjectivity of the interpretation of US images, the intra- and inter-rater reliability of this schema will also need to be assessed in follow-up studies. As larger studies are done, we may be able to identify semantic features for identifying various grades of glial tumors. Further correlation with MRI semantic criteria (VASARI) and more histological data will help to verify the applicability of ultrasound semantic criteria in predicting various histological features. Also linking criteria to an ontology network will help future research. Although its value with respect to changing operative decisions is presently limited, eventually technology may evolve so that ultrasound may be able predict tumor characteristics during (and maybe even prior to) the surgery. Therefore, systematic collection of data will help us build this information database. Also, in years to come, with large data and correlation with other patient parameters, semantic features may help predict response to therapy and prognosis. Even though interpretation of tumor histology may not have a direct impact on the course of surgery immediately, a uniform description of tumor delineation can help to improve the interpretation of tumor remnants. More importantly, it can help standardize reporting of residue across different studies, thereby making comparisons of extent of resection (based on ultrasound assessments) more reliable. This is important to validate the diagnostic accuracy of ultrasound. We acknowledge that the schema appears to be extensive and may not be easily applicable in routine practice. However, we strongly believe that a standardized recording of findings are crucial especially as ultrasound images are not routinely archived. In the setting of research studies, such standardized reporting formats are very important. Finally, in the era of big data and artificial intelligence, standardized terminology, and annotation are indispensable for utilizing precious resources such as ultrasound imaging data.

## Conclusion

We present a comprehensive semantic feature-based classification schema for ultrasound features in gliomas and have validated it in a cohort of high-grade gliomas. We hope to expand on the scope of its application to other brain tumors and refine the schema to be able to serve a crucial role in the future.
