# Supplementary material for: Development of a Standardized Semantic Feature-Based Reporting Proforma for Intraoperative Ultrasound Findings in Brain Tumors and Application in High-Grade Gliomas – A Preliminary Study
Source: Ultrasound Int Open. 2021 Nov 17;7(2):E55–63. doi: 10.1055/a-1637-9550 (PMC8598392; doi:10.1055/a-1637-9550)
Supplement: Supplementary file 1 — Supplementary Material [file 10-1055-a-1637-9550-0228.pdf]

Supplementary Material - Appendix 1

Ultrasound semantics features-based proforma for intraoperative ultrasound reporting of brain tumors

The proforma is based on the following five sub-components

1. Image quality

2. Anatomical landmarks

3. Overall features

4. Lesion Characteristics

5. Perilesional zone characteristics

Feature	Explanation	Descriptors	Remarks
Image quality	This describes the overall image quality and takes into account the adequacy of coverage (tumor and surrounding landmarks) as well as the presence or absence of artifacts	i. Good<br>ii. Moderate<br>iii. Poor	Good quality is when the lesion is adequately covered along with at least 1-2 landmarks and without significant artifacts. Moderate quality is when either the lesion or landmarks are not well covered with minimal artifacts. Poor quality is reported when either the lesion is poorly covered or there are significant artifacts degrading the image and making further interpretation impossible.
Anatomical landmarks	Descriptor of all visualized anatomical landmarks	Includes (but not limited to)<br>Falx, tentorium, base of skull, ventricles, choroid plexus, vessels, prominent fissures/sulci, etc.	-

Overall features			
Visualization	Defined as the ability of US to identify a lesion	i. Easily discernible<br>ii. Discernible with difficulty<br>iii. Not discernible	(i) indicates lesions that can be seen with relative ease, (ii) with difficulty, whereas (iii) indicates a lesion that cannot be seen on US.
Demarcation of lesion and perilesional zone	Are the two appreciable	I. Yes<br>II. No	If separate, both to be described as below. If not, entire visualized abnormality will be considered as the lesion and described as such. No prelesional zone can be described in such cases.
Entire lesion seen		I. Yes<br>II. No	This denotes whether the entire lesion is visualized or not
Lesion Characteristics			
Margin delineation	Defined by the extent of margin that can be clearly delineated from the perilesional zone/normal brain within the insonated circumference. Sometimes the US image may not encompass the entire circumference of the lesion. In that case the image section showing the maximum lesion to be used to assess this.	i. Good<br>ii. Moderate<br>iii. Poor	Good (more than 90% of circumference visualized well)<br>Moderate (>50% but less than 90% circumference visualized well)<br>Poor (< 50% circumference visualized well)

Margin type	Defined by the shape of the lesion and type of margin. Deep brain-tumor interface is more important than the surface which may sometimes not be optimally scanned. For this feature to be recordable, margin delineation (feature above) should be at least good or moderate. Otherwise it is recorded as (iv) cannot be characterized.	I. Regular, smooth<br>II. Regular, lobulated<br>III. Irregular crenated<br>IV. Cannot be characterized	I is used when the lesion is completely smooth and rounded. If it is smooth with lobulations, it should be recorded as II. III denotes lesions with irregular and/or spiculated margins extending into the surrounding space.
Lesion size	Measured in the 3 largest dimensions (length, breadth, and height)	l, b, h	If some part of the lesion boundary is obscured, the margin can be assumed by extrapolation to calculate the dimension if needed.
Echotexture	Described with respect to surrounding brain parenchyma (white matter which is typically hypoechoic)	I. Hyperechoic<br>II. Isoechoic<br>III. Hypoechoic<br>IV. Anechoic	(Self-explanatory)
Internal heterogeneit y	Characterizes the uniformity (or lack of it) of the internal tissue echogenicity	I. Homogeneous<br>II. Heterogeneous	Presence of cysts, necrosis, calcifications will all contribute to the heterogeneity
Presence of cyst	Defined as well demarcated hypo-/anechoic component. Usually, will show rim enhancement on the far aspect.	I. None<br>II. Single<br>III. Multiple	(Self-explanatory)
Cyst location	Describes the location of the cyst/s.	I. Intralesional<br>II. Perilesional<br>III. NA	If cyst is within the lesion margins, it is classified as intralesional.
Presence of necrosis	Defined as intralesional, irregular heterogeneous component with internal double densities/ – usually	I. None<br>II. Single<br>III. Multiple	May sometimes be admixed with cysts and calcifications

	hyperechoic but with variable areas of hypoechogenicity interspersed		
Amount of necrosis	Semiquantitative estimate relative to the main tumor mass	I. Absent<br>II. <10%<br>III. 10-50%<br>IV. >50%	
Presence of calcifications	Defined as extremely hyperechoic focal intralesional elements with posterior acoustic shadowing	I. None<br>II. Single<br>III. Multiple	May be seen in cases of hemorrhage (but usually posterior shadow is seen in calcification only)
Perilesional zone			
Perilesional zone characteristic	Should be separately identifiable from the main tumor. This perilesional area may represent infiltrating tumor, edema, gliotic brain, or a combination. Describe it relative to the tumor echogenicity.	I. None<br>II. Hyperechoic only<br>III. Both hypo- and hyperechoic components	(iii) a thin hypoechoic zone separates the tumor boundary from the more peripheral hyperechoic perilesional zone.
Perilesional zone margin type	This describes the type of the perilesional zone.	I. Regular<br>II. Irregular/ crenated/ finger-like<br>III. Cannot be characterized<br>IV. NA	If margin is not encompassed in the scan, it should be recorded as (iii) "Cannot be characterized".<br>In case there is no separate zone it should be recorded as (IV) NA
Extent of perilesional zone	Defines the extent relative to the main tumor mass	I. None<br>II. Less than size of tumor<br>III. Equal to size of tumor<br>IV. More than tumor<br>V. Cannot be assessed (NA)	In case there is no separate zone OR its margin is not encompassed in the scan, it should be recorded as (v) "Cannot be assessed"
Posterior insonation	Describes the artifacts specifically related to the	I. Enhancement<br>II. Shadowing<br>III. Mixture/both	Enhancement is the intense hyperechoic

effects/ artifacts	posterior acoustic phenomena which can help characterize some of the internal structures	IV. None	rim seen just beyond an anechoic (cyst) component<br><br>Shadowing is the dark/black zone beyond a densely hyperechoic internal echo (usually associated with calcification)
Other findings	Please record any other findings not mentioned above		

* US - Ultrasound
